# Mind the gender gap: how defining head trauma increases reporting in women aged 30–50

**DOI:** 10.3389/fneur.2025.1609098

**Published:** 2026-06-18

**Authors:** Christopher Fifty, Sarah Mayes, Benjamin Ehret, Katelyn Haeffner, Daniel Seichepine

**Affiliations:** Department of Life Sciences, Neuropsychological Laboratory, University of New Hampshire, Manchester, NH, United States

**Keywords:** mTBI, TBI, women, gender, midlife, neuropsychology, concussion, multiple

## Abstract

**Background:**

The use of an objective measure to evaluate the history of head trauma has been shown to significantly impact the amount of reported sustained head trauma in athletic male populations. However, the generalization of this definition across a more diverse population remains challenging; the World Health Organization notes that 93% of current research on mild traumatic brain injuries lacks gender-specific data. Additionally, compared to reports on young adults, there is limited research on the effects of concussions in midlife. Researchers hypothesize that women aged 30–50 from a general population would report additional head injuries when provided with an objective definition similar to those previously used for male athletes.

**Materials and methods:**

Highly educated women (M = 16.7 years of education, SD = 2.1) aged 30–50 (M = 40.3, SD = 5.1) completed an online survey. The survey collected demographic data on sports participation and head trauma history. Race, ethnicity, and socioeconomic status were not collected. Participants first reported lifetime mTBI history without an objective definition, and were then reassessed after being given an objective definition.

**Results:**

A Wilcoxen Signed Rank test indicated a significant change in number of reported head injuries, pre and post definition (*z* = 4.06, *p* < 0.0001). The number of head injuries reported increased for 42% of the population, for this portion of the sample the median increased fourfold. To better understand the differences between those who reported an increase, and those who did not, researchers performed an examination of commonalities between those groups. A Chi-Square Test of Independence indicated a significant relationship (chi = 7.03, *p* < 0.01) between participation in sports (recreational, organized) and change in reported head injuries: 21% of individuals without a sport history increased the number of reported head injuries, in contrast 58% of individuals with a sport history increased the number of head injuries reported.

**Discussion:**

Given the significant increase observed in reported mTBIs, this definition is a robust tool that may help researchers and clinicians, more accurately identify lifetime head trauma in women. Similar to findings in male athletic populations the increase in number of reported mTBIs was significantly greater in women with an athletic history. Through more accurate reporting this definition could help improve clinical care as well as research into better understanding the impact of head injuries. Further research into the effects of race, ethnicity, and socioeconomic status, in relation to gender and mTBI history, could serve to address opportunities related to the generalizability of this study.

## 1 Introduction

Each year in the U.S., as many as 69,000 individuals die as a result of (or due to complications stemming from) a traumatic brain injury (TBI) (1). TBI is a broad classification used to categorize injuries that result in structural, and or functional changes to the brain (2). TBI severity spans a continuum, from mild to severe. Identifying mild traumatic brain injuries (mTBIs) is challenging because of underreporting due to variability in presentation, which limits common understanding of what constitutes an mTBI (3). Further overlapping symptoms might be assumed to be comorbid (or better attributed) with other conditions (4). Despite difficulty related to assessing prevalence, mTBIs are believed to account for as much as 75% of all TBIs (5).

mTBIs occur when an impact to the head or body results in temporary neurological symptoms such as headache, confusion, sensitivity to light or sound (6). While most individuals recover within 2 weeks, some experience symptoms that persist beyond 3 months, leading to chronic physical, neuropsychiatric, and cognitive impairments that can significantly impact quality of life. Research over the past decade has demonstrated a link between repeated mTBIs and long-lasting structural and functional changes in the brain, resulting in long-term social and occupational difficulties (7). In certain populations, symptoms may worsen with repeated injuries (4). This type of long-term cognitive impairment from successive mTBI exposure, may result in an increased risk of dementia following mTBI exposure later in life (8). Furthermore, growing evidence shows that for some individuals, consequences of mTBI may lead to notable behavioral impairments and unstable housing or employment, and in some cases incarceration (9). Acquiring a mTBI has demonstrated an increased likelihood of incurring additional mTBIs, and as a result producing long-term functional deficits (7).

Research on mTBI has traditionally focused on athletic populations due to the frequency of the injuries in contact sports, and the ability to identify them shortly after they occur. The utilization of accelerometers has provided considerable insight into the frequency of head injuries within this population (10). Results have shown to range from several hundred head impacts to well over 1,000 over a span of an athletic season. Further, for some athletes who had never endorsed a history of mTBIs, postmortem evaluations have shown structural changes consistent with chronic traumatic encephalopathy, suggesting that injuries without neurological symptoms can cause significant neurological damage (29). A recent review of TBI-related literature suggests outcomes lean worse for women than for men (11). This contradicts earlier animal research, which indicated that mTBI outcomes were typically better for females, including less cognitive impairment in females, and reduced anxiety under certain conditions (12, 14). These findings highlight the need for continued human subjects research to better understand gender disparities in TBI outcomes.

Despite this need, research focusing on women in TBI related literature, or even providing gender-related data, remains largely an anomaly (14). The World Health Organization (WHO) reported that 93% of the TBI literature did not specify gender (15). The American Congress of Rehabilitative Medicine issued a call to action in 2010 to address this disparity. However a recent scoping review published in 2023 found no change in the average number of studies published per year into the effects of TBIs in women, following this call to action (13). Research that has been conducted in mTBI populations has suggested staggering gender-specific outcomes in women including: worse performance on neurocognitive testing, experiencing more symptoms, a sharper decline in executive functioning 6 months post-TBI, and a greater impairment in ocular reflexes 3 weeks post-TBI (15–18), TBI related research has historically suggested that most symptoms following an mTBI resolve within 3 months following the injury, except in cases of persistent post-concussion syndrome (18). More recent research suggests that up to half of individuals who suffer from an mTBI may experience long-term cognitive impairment (8). Given that the literature indicates women experience more symptoms following an mTBI, they may also be at greater risk for chronic symptoms. Furthermore, TBI related literature into middle-aged populations is limited, with most studies focusing on adolescent, athletic, or geriatric populations (19). Limited prevalence data has suggested, that of mTBIs that are treated in hospital settings, the median age of an individual seeking treatment is approximately 40 years of age (20). This highlights the need for a clinically appropriate measure to evaluate lifetime trauma in women between the ages of 30 and 50.

A lack of a clear definition for assessing mTBI in women has likely contributed to underreporting, and possibly leading to poorer clinical outcomes. This study applied a previously validated definition of mTBI that has been shown to increase injury reporting in male populations (21–23, 27). Our aim was to determine whether the same definition would lead to a similar increase in reported mTBI cases among women aged 30–50.

## 2 Materials and methods

### 2.1 Participants

Fifty self-identified females were recruited through a combination of social media and word-of-mouth. Participants were required to speak English, to be between 30 and 50 years of age (M = 40.3 years, SD = 5.1 years). Most participants had a graduate degree (54%), followed by a bachelor’s degree (24%), while 22% were without a bachelor’s degree.

### 2.2 Measure and procedure

After recruitment, participants were directed to an online, Qualtrics study, that took approximately 45 min to complete. Participants were not required to complete the survey in one sitting, and could leave and return to the study at their leisure. Additionally, Qualtrics made the study accessible on most devices including mobile phones, laptops, and tablets. Upon completing the study, participants received a $15 Amazon gift card. Participants reported the number of head injuries experienced over their lifetime, both before and after being provided a definition of mTBI. Given that TBI research has shown a high prevalence of TBIs in individuals who play contact sports, the survey also assessed the frequency of participation in activities commonly linked to mTBI (e.g., organized/recreational sports, military experience, motor vehicle accidents). Individuals were also assessed for a history of medical attention, in relation to TBI. Data was collected on whether or not participants had seen a doctor following a hit to the head. Race, ethnicity, and socioeconomic status were not collected.

### 2.3 Number of head injuries

Participants were probed twice for their self-reported history of head injuries. The first time, participants were asked for a summary of lifetime trauma with the following verbiage:

“As best as you can remember, approximately how many total head injuries have you had during your lifetime?”

Following their answer to this prompt, participants were asked to provide demographic information, and were then given the following prompt:

“Some people have the misconception that head injuries only happen when you black out after a hit to the head or when the symptoms last for a while. But, in reality, a head injury has occurred anytime you have had a blow to the head that caused you to have neurological symptoms for any amount of time. These include: blurred or double vision, seeing stars, sensitivity to light or noise, headache, dizziness or balance problems, nausea, vomiting, trouble sleeping, fatigue, confusion, difficulty remembering, difficulty concentrating, or loss of consciousness. Whenever anyone gets a “ding” or their “bell rung,” that too is a head injury.

Based on that definition of a head injury, as best as you can remember, approximately how many total head injuries have you had during your life?”

## 3 Results

**Figure 1:**
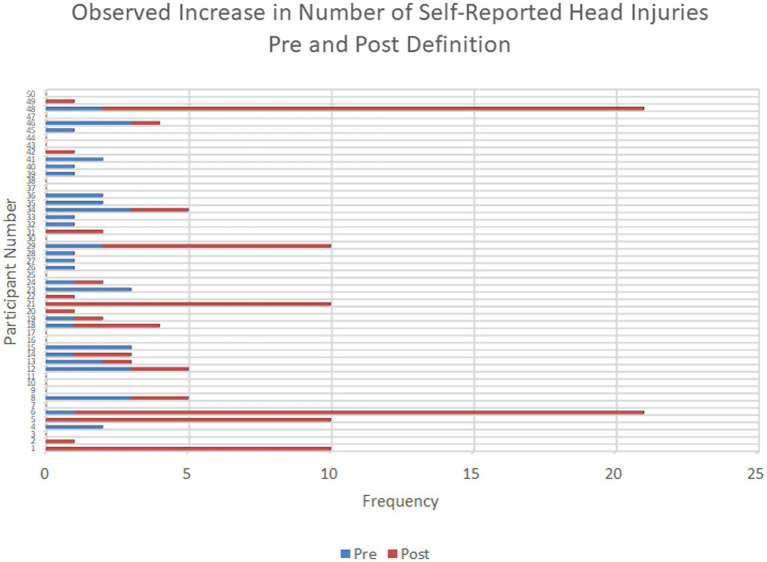
Observed increase in number of self-reported head injuries pre and post definition, per participant.

A Wilcoxen Signed Rank test indicated a statistically significant change in the number of reported head injuries, pre and post definition (*z* = 4.06, *p* < 0.0001). The mean number of reported head injuries pre and post definition were 0.9, and 2.88, respectively. The number of reported head injuries increased for 42% of the sample, with the mean reported increasing fourfold (see Figure 1).

To better understand demographic differences between those who reported an increase, and those who did not, an evaluation of differences between groups was performed. A Chi-Square Test of Independence indicated a significant relationship (chi = 7.03, *p* < 0.01) between participation in sports (recreational or organized), and a change in reported head injuries. Of those individuals without a sports history, only 21% increased the reported amount of head injuries post definition. For those who identified participation in sports, however, 58% showed an increase. Of the 26 individuals who reported a history of at least 1 head injury prior to the use of the objective definition, 65% reported seeing a doctor following that head injury. 86% of participants completed the survey in less than an hour, with a median duration of 22.1 min, with a range of 9–4,549 min. 28% of participants elected not to provide the age of last injury, but those who reported had a mean number of years since last injury of 13.8, with a standard deviation of 11.9 years.

## 4 Discussion

The significant increase in reported head injuries among participants when provided with a robust definition highlights the value of this tool in both research and clinical settings. Women who report higher numbers of head injuries could benefit from targeted monitoring to identify early signs of chronic conditions. Incorporating this definition as a standardized question during routine medical appointments could improve the accuracy of head injury histories, facilitating early prevention and symptom management. Notably, routine medical checkups offer a meaningful opportunity to screen for lifetime head injuries, especially in those who may not have realized that their past experiences constitutes an injury. Further, rehabilitation clinics could also benefit from a standardized low-cost screening tool to track mTBI history, to better assist in accurate treatment planning with populations where assessment tools remain limited. By integrating a relevant definition into the intake process and electronic health records, providers could gather more accurate medical histories, and potentially improve overall long-term patient care. Additionally, this tool could serve an educational purpose, helping individuals understand the importance of their head trauma history and encouraging timely clinical evaluations when symptoms develop. Knowledge to help identify their experiences, can reduce the likelihood of preventable symptoms of occurring in the future.

A lack of a clear definition for assessing mTBI in women has likely contributed to underreporting, and possibly leading to poorer clinical outcomes (24). This study applied a previously validated definition of mTBI that has been shown to increase injury reporting in primarily male populations (21–23). Our aim was to determine whether this definition would lead to a similar increase in mTBI cases among women aged 30–50. Similar to results in male populations, when provided with the aforementioned definition, women reported significantly greater amounts of head injuries. The results further demonstrated that individuals with a history of organized or recreational sports, were on average more than two and a half times as likely to report an increase in the amount of lifetime head injuries (21% of participants without, vs. 58% of participants with a history of participation in sports).

Integrating gender-specific research into mTBI definitions is crucial for advancing clinical care. Research demonstrates that women often exhibit distinct symptoms, such as prolonged post-concussive issues and emotional dysregulation, which are less commonly reported by men (25). Current diagnostic criteria for mTBI predominantly based on male populations, may not capture the full spectrum of women’s experiences, particularly those exposed to high-risk environments. This gap can lead to underdiagnosis or delayed treatment in female patients. By refining the diagnostic method of assessing mTBI history, clinicians could better tailor diagnostic and treatment protocols. Such improvements would enhance personalized care, reduce misdiagnosis, and ensure more effective management of symptoms in women, particularly those involved in high-risk activities. This approach also highlights the need for inclusive research and gender-sensitive therapeutic interventions to improve long-term outcomes.

With adoption, this definition could afford clinicians the ability to better understand one’s lifetime history of mTBIs. Research has demonstrated, increasingly, that as the number of sustained mTBIs increases, so too can the consequences leading from these injuries. In women this can translate to an increased risk of developing dementia later in life, including hormonal dysregulation, such as pituitary dysfunction and hyperprolactinemia, which can disrupt menstrual cycles and fertility (26). Additionally, women with repeated mTBIs are at a heightened risk for chronic migraines, and mood disorders like depression and anxiety (25). Utilizing this definition could further improve researcher’s ability to evaluate consequence of repetitive mTBIs within a population characterized by a relative lack of research, relative to geriatric, and adolescent populations.

The generalizability of these findings are limited by a variety of factors. The sample for this study was comprised of 50 women, through a sample of convenience. Researchers did not collect data related to participants race, ethnicity, or socioeconomic status which limits the ability of these results to be generalized to other populations The study was rendered through Qualtrics, a platform that afforded participants the ability to take the test through many technological mediums (including phones, tablets, and laptop computers), data related to the method participants used to take the study was not tracked or recorded. No environmental control was available for this study, and participants were allowed to leave the session, and to return to it, across multiple settings. Lastly, duration was not standardized for our sample, therefore no information was recorded when participants chose to stop or resume the survey.

Previous research has demonstrated that education increases the likelihood that individuals will report better health outcomes. Likewise, lower education is linked to worse cognitive dysfunction following mTBI.50 Given that over half of the sample had a graduate level degree, this could perhaps limit the generalizability of the observed effect, to a general population. In an effort to improve the generalizability of the research, and through what researchers believe should be a continuous process, the authors recommend that information related to race, ethnicity, and socioeconomic status be factored into account in the future.

The body of mTBI related literature has demonstrated significant disparities with relation to age and gender. This study has demonstrated that a definition, previously shown to be effective in male populations, can now be used more robustly, without prejudice to middle-age or sex.

## Data Availability

The datasets presented in this article are not readily available because the consent document (signed by the study participants) stipulated that data would not be shared with any individual or organization. Requests to access de-identified data should be directed to the corresponding author.
